# Stearyl amine-modified elastic cerosomes for boosting the anti-cancer activity of albendazole

**DOI:** 10.3389/fphar.2025.1595177

**Published:** 2025-09-04

**Authors:** Rofida Albash, Hassan M. E. Azzazy, Shaimaa Mosallam, Mohammed I. A. Hamed, Khaled M. Darwish, Maha M. Abdel-Fattah, Lama A. Alshabani, Einas M. Yousef, Heba Mohammed Refat M. Selim, Ghadeer El-Fadaly, Asmaa Saleh, Diana E. Aziz

**Affiliations:** ^1^ Department of Pharmaceutics, College of Pharmaceutical Sciences and Drug Manufacturing, Misr University for Science and Technology, Giza, Egypt; ^2^ Department of Chemistry, School of Sciences and Engineering, The American University in Cairo, New Cairo, Egypt; ^3^ Department of Pharmaceutics and Industrial Pharmacy, Faculty of Pharmacy, October 6 University, Giza, Egypt; ^4^ Department of Organic and Medicinal Chemistry, Faculty of Pharmacy, Fayoum University, Fayoum, Egypt; ^5^ Department of Medicinal Chemistry, Faculty of Pharmacy, Galala University, New Galala, Egypt; ^6^ Department of Medicinal Chemistry, Faculty of Pharmacy, Suez Canal University, Ismailia, Egypt; ^7^ Department of Pharmacology and Toxicology, Faculty of Pharmacy, Beni-Suef University, Beni Suef, Egypt; ^8^ Department of Pharmaceutical Sciences, College of Pharmacy, Princess Nourah bint Abdulrahman University, Riyadh, Saudi Arabia; ^9^ College of Medicine, Alfaisal University, Riyadh, Saudi Arabia; ^10^ Department of Pharmaceutical Sciences, College of Pharmacy, Almaarefa University, Dariyah, Saudi Arabia; ^11^ Department of Pharmaceutics and Industrial Pharmacy, Faculty of Pharmacy, Cairo University, Cairo, Egypt

**Keywords:** albendazole, repurposing, elastic cerosomes, stearyl amine, solid ehrlich

## Abstract

**Background:**

Albendazole (ALB), originally developed as an anthelmintic agent, has been repurposed for use in cancer therapy. In the present work, ALB was incorporated into stearyl amine (SA)-based elastic cerosomes (EC) to improve its anticancer activity.

**Methods:**

Stearyl amine elastic cerosomes containing albendazole (SA-EC-ALB) were formulated using the thin-film hydration method. A D-optimal experimental design was applied via Design-Expert® software (version 7) generating 19 formulations. The independent variables included SAA amount (X1), sonication time (X2), ceramide type (X3), and SAA type (X4), while the dependent variables were entrapment efficiency (EE%; Y1), particle size (PS; Y2), and polydispersity index (PDI; Y3).

**Results:**

The optimized SA-EC-ALB formulation, prepared with ceramide III and Pluronic L121 through sonication, achieved an entrapment efficiency (EE%) of 92.03 ± 3.53% and a particle size (PS) of 312.05 ± 9.32 nm. *In-silico* analysis indicated strong interactions between ALB and the vesicular components in water. Moreover, *in-vivo* evaluation of SA-EC-ALB antitumor activity, performed using the solid Ehrlich tumor model in adult Swiss albino male mice, demonstrated a significant reduction in tumor volume compared to the control group.

**Conclusion:**

Loading ALB into SA-EC could potentially induce its anticancer effects.

## 1 Introduction

Cancer is one of the major public health issues that ranks high as a leading cause of death globally (Song et al., 2022) It is associated with abnormal cell proliferation and absence of proper cell cycle regulation and apoptosis (Siddiqui et al., 2022). Due to uncontrollable metastasis, the disease prevalence increased with a concomitant reduction in the treatment effectiveness (Siddiqui et al., 2022). Despite the development of targeted anti-cancer drug therapy, the patients’ survival rates are still low as a consequence of the swift emergence of drug resistance, poor vascularization and hypoxia of the cancer cells (Siddiqui et al., 2022; Song et al., 2022). Accordingly, there is a strong demand for the development of new anti-cancer therapeutics that can overcome drug resistance and prevent metastasis.

Development of new anti-cancer drugs includes many pharmacological tests, pre-clinical and clinical studies which require high costs and prolonged development times. In this context, drug repurposing—or reprofiling—has been recognized as a promising strategy to uncover alternative therapeutic applications for drugs in current clinical use (Olgen and Kotra, 2019). It is an attractive alternative strategy in the field of drug development thereby circumventing the elaborate steps and considerable costs inherent in standard drug development. Repurposing of existing drugs with known drug profile gives a better understanding of their pharmacodynamics, pharmacokinetics and metabolic profiles (Siddiqui et al., 2022). Recently, drug repurposing for cancer treatment has been attracting attention to avoid well-known drug resistance issues and to customize treatment plans that minimize adverse effects in cancer patients (Zhang et al., 2020).

Albendazole (ALB) is one of benzimidazole anthelmintic drugs acts by inhibition of microtubules polymerization by destructing *β*-tubulin structure. It also causes depletion of glycogen stores with lowering ATP formation in susceptible parasites by blocking glucose uptake. As a result, it contributes to immobilization and death of the worms (Castro et al., 2016; Liu et al., 2020). Due to its microtubule’s interaction, ALB recently has been showing great potential as repurposed anti-cancer drug. Its repurposed anti-cancer efficacy is augmented by other mechanisms including; inhibition of metastasis, induction of apoptosis and cell-cycle arrest (Song et al., 2022). Furthermore, ALB can sensitize tumor cells to ionizing radiation which enlightens its use for brain metastasis in combination with radiotherapy (Patel et al., 2011). According to reports, it is effective against a wide range of cancer cells. e.g., prostate cancer, ovarian cancer, colorectal cancer, and hepatocellular carcinoma cells (Pourgholami et al., 2001; Ehteda et al., 2012; Noorani et al., 2014; Liu et al., 2020).

Recently, breast cancer has emerged as one of the most prevalent cancers among women globally. Breast cancer therapeutic strategies are based on surgery, radiotherapy and chemotherapy (Radulski et al., 2023). The currently available anti-breast cancer drugs are very costly and show low efficacy and high toxicity with high rates of cancer progression and recurrence (Oliveira et al., 2023). Furthermore; heart problems, infertility, blood clots and bone loss are common side effects of anti-breast cancer drugs that are difficult to be tolerated by cancer patients (Aggarwal et al., 2021). Hence, utilizing ALB as repurposed drug for breast cancer is expected to be a promising solution for bypassing these issues (Song et al., 2022). However; ALB, being a BCS class II compound (water solubility (0.2 µg/mL)), suffers limited systemic exposure and erratic bioavailability after oral administration (Racoviceanu et al., 2020; Shah et al., 2023). Therefore, the poor water solubility and oral bioavailability (less than 5%) are considered as obstacles against the clinical use of ALB as anti-cancer drug. Several researches have been conducted with a main goal of enhancing ALB aqueous solubility by formulating it as solid-lipid nanoparticles (Marslin et al., 2017), chitosan-PLGA nanoparticles (Kang et al., 2017), albumin nanoparticles (Noorani et al., 2014), polyurethane nanoparticles (Racoviceanu et al., 2020) which have shown promising results on various cancer cell lines. The use of advanced topical nano-formulations of ALB, e.g., cubosomes and ufasomes is considered as a talented approach for bypassing ALB poor oral bioavailability and enhancing its skin permeation and retention (Saber et al., 2021; Abedin et al., 2022).

Recently, the investigation has been focusing on developing cerosomes (ceramide-based tubular vesicles) using phospholipids and different surfactants (SAA) (Eltabeeb et al., 2025; Mohamed et al., 2025). Ceramides are main components in sphingolipid biosynthetic pathway with diverse tumor suppressive effects. Therefore, utilizing ceramides for cancer treatment is considered key target for study and intervention (Galadari et al., 2015). Incorporation of surfactants in cerosomes formulations could enhance the vesicular stability and prolong their residence with consequent better drug efficacy (Vega et al., 2013). Abdelgawad R. et al., utilized ceramide VI, phospholipids and different SAA for efficient treatment of psoriasis using Tazarotene (Abdelgawad et al., 2017). Furthermore, Albash R. et al., utilized PEGylated cerosomes and hyaluronic acid enriched cerosomes for enhancing skin delivery of Fenticonazole and Spironolactone respectively. Up-to-date, poloxamers modified cerosomes have not been yet investigated. Poloxamers (Pluronics) are amphiphilic triblock copolymers that when incorporated with phospholipid vesicles can act as vesicular stabilizers, enhance the micro-mechanical properties of the vesicular bilayer and consequently prolong the drug release rate from vesicles (Liang et al., 2005).

Due to rapid cell division and accumulation of excessive lactic acid, cancer cell surface showed significantly higher negative charge than normal tissues. Therefore, utilizing positively charged nanocarriers is hypothesized to target cancer cells selectively, differentiating them from normal tissues (Vishwakarma et al., 2024).

Hence, our research is conducted for attaining two main goals; firstly, formulating ALB, as a repurposed anti-cancer drug, elastic cerosomes (EC) (ALB-EC) a type of flexible lipid-based nanocarrier using different Pluronics with different hydrophilicity according to D-optimal design with the aid of Design-Expert^®^ software to investigate the impact of different variables on vesicular characteristics and to suggest the optimal EC-ALB based on desirability function. Secondly; to study the influence of incorporation of positively charged lipids (SA) on cerosomes’ physico-chemical characteristics, EC-ALB was used as nucleus for formulating stearyl amine elastic cerosomes loaded Albendazole (SA-EC-ALB) by using various amounts of SA. The optimal amount of SA was then selected and used for preparation of SA-EC-ALB which was then assessed for its morphology using transmission electron microscopy (TEM). *In-silico* study was also performed to anticipate the interaction between ALB and other ingredients in different media. Finally, *in-vivo* tumor volume (TV) assessment and histopathological studies were conducted in adult *Swiss albino* male mice using solid Ehrlich tumor model aimed at examining both the tumor-inhibiting potential and the safety characteristics of SA-EC-ALB formulation.

## 2 Materials and methods

### 2.1 Materials

Albendazole (ALB) was granted by Alexandria Co. (Cairo, Egypt). L-α phosphatidylcholine (PC), Pluronic L121 and Pluronic P188 were acquired from Sigma-Aldrich (St. Loius, MO, United States). Ceramide III and ceramide IIIB were purchased from Evonik Co. (Essen, Germany). Stearyl amine (SA) was acquired from Fluka Chemical Co. (Buchs, Switzerland). Chloroform and Methanol were procured from El-Nasr Pharmaceutical Chemicals Co. (Cairo, Egypt).

### 2.2 Fabrication of elastic cerosomes loaded albendazole (EC-ALB)

Elastic cerosomes loaded Albendazole (EC-ALB) were developed through the use of thin film hydration technique (Ahmed et al., 2024) Briefly, ALB (2 mg), PC (100 mg) together with variable amounts of Pluronics (L121 or P188) and ceramides (III or IIIB) were weighed and dissolved in 10 mL chloroform in round-bottom flask (250 mL). The organic solvent was evaporated under vacuum using a rotatory evaporator (Rotavapor, Heidolph VV 2000, Burladingen, Germany) that rotated for 30 min at 90 rpm and 60 °C. After that, 10 mL of ultra-pure distilled water was used to hydrate the resulting clear film for 45 min at 60 °C under normal pressure. Finally, to avoid aggregation, the formed elastic cerosomesʼ dispersions were subjected to varying durations of sonication (10, 20 or 30 min) at 25 °C using ultra-sound bath (Model SH 150-41; United States) (Joseph et al., 2020).

### 2.3 Experimental design

To evaluate the individual and combined effects of the formulation variables on the characteristics of the nanosystems, EC-ALB formulations were prepared using a D-optimal design generated by Design-Expert® software (version 7, Stat-Ease, Inc., Minneapolis, MN, USA). A total of 19 formulations were prepared according to the experimental design. The independent variables were SAA amount (X1), sonication time (X2), ceramide type (X3), and SAA type (X4), while the dependent variables included entrapment efficiency (EE%; Y1), particle size (PS; Y2), and polydispersity index (PDI; Y3). The best-fitting mathematical model was selected to obtain the highest predictive R^2^ value (Eldeeb et al., 2019). The ranges of the studied factors and the constraints applied to the responses are summarized in Table 1.

**TABLE 1:** D-optimal design parameters and target constraints used for optimization of EC-ALB.

Factors (independent variables)	Lowest level	Highest level
X_1_: SAA amount (mg)	50	75
X_2_: Sonication time (min)	10	30
X_3_: Ceramide type	Ceramide III	Ceramide IIIB
X_4_: SAA type	Pluronic L121	Pluronic P188

Responses (dependent variables)	Desirability constraints
Y_1_: EE%	Maximize
Y_2_: PS (nm)	Minimize
Y_3_: PDI	Minimize

Abbreviations: SAA, surface active agent; EE%, entrapment efficiency percent; PS, particle size; PDI, polydispersity index, and EC-ALB, elastic cerosomes loaded albendazole.

### 2.4 *In-vitro* characterization of elastic cerosomes loaded albendazole (EC-ALB)

#### 2.4.1 Determination of entrapment efficiency percent (EE%)

To determine the EE% of EC-ALB, the centrifugation method was chosen. Briefly, 1 mL of the produced dispersion was centrifuged for 1 h at a speed of 20,000 rpm at 4 °C using an ultra-cooling centrifuge (Sigma 3–30 KS; Sigma Laborzentrifugen GmbH, Osterode am Harz, Germany). Then, the concentration of the entrapped ALB was determined spectrophotometrically (Shimadzu UV1650 Spectrophotometer; Shimadzu Corp., Kyoto, Japan) after lysing the resultant sediment using methanol and analyzing it at the previously established ALB λ_max_ in methanol (295 nm) (Yan et al., 2024). Each measurement was performed in triplicates. This equation was used to determine the drug EE% (Ahmed et al., 2024).

$$EE\%=\frac{Incorporated\;amount\;of\;ALB}{Total\;amount\;of\;ALB}\times 100$$

#### 2.4.2 Determination of particle size (PS), polydispersity index (PDI), and zeta potential (ZP)

Dynamic light scattering implemented in Zetasizer Nano ZS (Malvern Instrument Ltd., Worcestershire, United Kingdom) was used to determine the mean PS, PDI, and ZP of the prepared EC-ALB. Before every measurement, de-ionized water was used to sufficiently dilute the produced dispersions until being hazy before analysis to obtain proper scattering intensity (Joseph et al., 2020; Aziz et al., 2022a). Each sample was measured three times.

### 2.5 Optimization of elastic cerosomes loaded albendazole (EC-ALB)

Predicting the optimal conditions for preparing the best formula which may not be included in the actual design is one of the most important advantages of D-optimal design (Eldeeb et al., 2019). Maximizing EE% and minimizing PS and PDI were the selected criteria for preparing the optimal EC-ALB formulation (Table 1). By applying desirability function, the optimal formulation was suggested, fabricated, *in-vitro* characterized, and the results were compared with software predicted responses. The predicted optimal EC-ALB was also used for more investigations.

### 2.6 Preparation of optimal SA-EC-ALB

SA-EC-ALB formulation was prepared utilizing the same ingredients as the optimal EC- ALB along with varying amounts of SA (5, 10, 15 mg). The formula was fabricated utilizing the same thin film hydration technique applied to fabricate EC- ALB, and SA was also dissolved in the chloroform. The prepared SA-EC-ALB formulations were evaluated for EE%, PS, and ZP. The results were analyzed statistically using one-way ANOVA in SPSS software (version 17.0, SPSS Inc., Chicago, IL), followed by Tukey's HSD post-hoc test. A p-value of less than 0.05 was considered statistically significant. The optimal SA concentration was selected for the preparation of SA-EC-ALB and subsequently subjected to further *in-vitro* and *in-vivo* studies.

### 2.7 *In-vitro* release

The total amount of drug released after 6 h (Q6h) was calculated utilizing the United States Pharmacopeia (USP) dissolution apparatus (Pharma Test, Hainburg, Germany) for 6 h at 37 °C. An amount of 2 mL samples from SA-EC-ALB, EC-ALB and ALB aqueous suspension (each containing 200 µg ALB) were enclosed in cylindrical plastic tube (3.14 cm^2^ permeation area). One end of these tubes with one end securely sealed with a cellulose membrane (Spectra/Por^®^ 12,000–14,000 molecular weight cutoff, Spectrum Laboratories Inc., USA) while the other one connected to the USP dissolution apparatus shafts instead of the baskets. The formulations were submerged in 50 mL of buffer solution with a pH of 7.4 (to maintain sink condition). At 1, 2, 3, 4, 5, and 6 h, aliquots were withdrawn (3 mL), and analyzed for the presence of ALB using UV spectrophotometer at λ_max_ 295 nm. In order to preserve sink conditions, an equivalent volume of fresh release medium was added immediately. The experiment was repeated three times. A schematic illustration of the release setup is provided in the Supplementary Material (Supplementary Figure S1) for better visualization. The following equation was used to determine the amount of ALB released.

$$\%\;Release=\frac{\;Amount\;of\;ALB\;released\;at\;atime\;t}{Total\;amount\;of\;ALB}\times 100$$

### 2.8 Transmission electron microscopy

The SA-EC-ALB was morphologically examined by TEM (Joel JEM 1230, Tokyo, Japan). On a carbon-coated copper grid, one drop of the SA-EC-ALB dispersion was deposited as a thin layer following staining with 2% w/v phosphotungstic acid. After drying, The sample was analyzed using a TEM at 80 KV (Ahmed et al., 2025).

### 2.9 Differential scanning calorimetry (DSC)

ALB and the optimal SA- EC- ALB were subjected to thermal investigation utilizing differential scanning calorimeter (Shimadzu DSC-60, Shimadzu Corp., Kyoto, Japan) that was calibrated with 99.9% purified indium. Each sample (about 5 mg) was placed in a conventional aluminium pan and heated between 10 °C and 300 °C at a scanning rate of 10 °C/min and a continuous nitrogen purging rate of 25 mL/min (Elmahboub et al., 2024).

### 2.10 Molecular docking studies

#### 2.10.1 Molecular modelling simulation of SA-EC-ALB

Computational analysis was conducted under vacuum conditions utilizing AutoDock software version 1.2.0 (free open–source software; Scripps Research Institute, California, United States). Drug and formulation additives were created in the two dimensions utilizing the isomeric SMILES that were deposited at PubChem database; albendazole (ID: 2082), ceramide-III (ID: 57378373), phospholipid (ID: 65167), stearyl amine (ID: 15793), and pluronic-L121 unit molecule (ID: 87350387). The structures were converted into three-dimensional conformations and energy-minimized under the AMBER force field with modified partial charge (O'Boyle et al., 2011). Molecular docking was carried out using the Lamarckian Genetic Algorithm with the AMBER force field to predict binding conformations (Eberhardt et al., 2021). Docking parameters were established at a maximum energy difference between poses of 4 Kcal.mol-1, global search exhaustiveness of 100, and binding poses of 20 (Xue et al., 2022). Docketed binding scores were given by software as binding energies (ΔG; Kcal.mol-1), whereas the optimal docked pose was chosen based on a combination of high docking scores and the presence of notable polar and hydrophobic interactions between the interacting formulation components.

The docked drug/nanoformulation complex served as the reference structure for all-atom MD simulations, prepared and inspected in VMD (Visual Molecular Dynamics, open-source); Theoretical and Computational Biophysics group, University of Illinois at Urbana-Champaign, Illinois, United States) (Humphrey et al., 1996). The drug-formulation complex was solvated using a 3D water cube-shaped box with marginal distances of 10 Å under periodic boundary conditions (Table 2). Standard ionization was set while as the model was neutralized via sufficient numbers of chloride and potassium ions. After the system was minimized for 50 ps using the NVT ensemble (300 K), it was equilibrated for 50 ps using the NPT ensemble (300 K; 1 atm. Pressure). Then, explicit molecular dynamics simulations were generated using 1,000 ps under NPT ensemble. At 200 ps intervals throughout the extracted timeframes, time-evolution conformational changes for the drug nano-formulation were observed.

**TABLE 2:** Atomic composition of drug nano-formulation for molecular dynamics simulation.

Solvation state	Atomic composition (No. of atoms)								
	ALB	PC	Ceramide-III	SA	Pluronic-L121	Aqua	K	Cl	Entire model
100% TIP3P water model	33	134	112	58	140	3 × 4,493	12	14	13.982

Abbreviations: ALB; albendazole, PC; phosphatidyl choline, and SA; stearyl amine.

### 2.11 *In-vivo* studies

The antitumor activity of SA-EC-ALB was assessed in a solid Ehrlich tumor model using adult male Swiss albino mice (n = 6) with an average weight of 22 ± 2 g. The group size of six mice per treatment group was determined based on ethical considerations aligned with the 3Rs (Replacement, Reduction, Refinement) principle, aiming to minimize animal use while ensuring scientific validity. Mice were kept in polypropylene cages in the animal house of the Faculty of Pharmacy, Beni-Suef University, in a light period of 12 h/day and at a temperature of 25 °C ± 2 °C and humidity of 50% ± 5%. The mice were randomly distributed into four groups. The study protocols followed the recommendations of the National Institutes of Health Guide for the Care and Use of Laboratory Animals (NIH Publication No. 8023, revised 1978). No consent from animal owners was required, as the study involved laboratory mice (Council, 2011). The group (1) was a normal control group and injected intraperitoneally (I.P) with vehicle solution. Groups (2-4) were injected with Ehrlich carcinoma cell line (0.2 mL/2–2.5 × 10^6^ cells/mouse) subcutaneously into the right thigh of each mouse to establish tumor in mice (Salem et al., 2018). The solid Ehrlich tumor in each mouse was developed after 7 days. Group 2 served as the positive control group while, groups 3 and 4 received ALB aqueous suspension and SA- EC- ALB (100 µg/mL) (Kang et al., 2015), respectively, in a dose of 2 mg/kg (440 µL), I.P/3 days/week for 3 weeks. The tumor volume (TV) was determined using digital caliber at the end of each week and after the last day of treatment administration, all mice were anesthetized with thiopental (30 mg/kg) and then sacrificed with cervical dislocation, and the right thighs were isolated for histopathological study. Tumor masses were isolated and fixed in 10% formol saline. Tumor tissue sections (5 μm thick) from the four groups were stained with hematoxylin and eosin (H&E) and examined histopathologically using a light microscope (Leica Microsystems GmbH, Wetzlar, Germany) (Bancroft and Gamble, 2008). GraphPad Prism software (Version 9.2.0.332 – San Diego, United States) was used for data analysis. Statistical significance is calculated using one-way ANOVA. Additionally, differences between the two groups are made using Tukey’s multiple comparisons test.

## 3 Results

### 3.1 Effect of formulation variables on EE% of elastic cerosomes loaded albendazole (EC-ALB)

**FIGURE 1:**
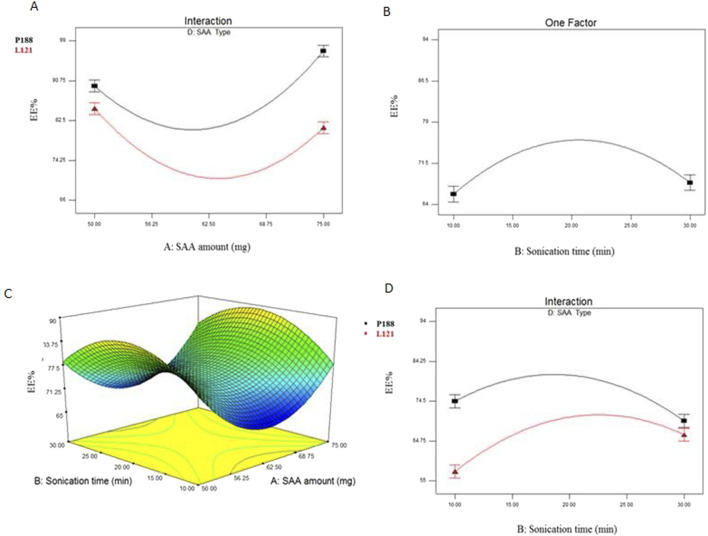
Interaction plot between SAA amount (X_1_) and SAA type (X_4_) **(A)**, line plot for the significant effect of sonication time (X_2_) **(B)**, response 3-D plot for the combined effect of SAA amount (X_1_) and sonication time (X_2_) **(C)** and interaction plot between sonication time (X_2_) and SAA type (X_4_) **(D)** on EE% of EC-ALB. Abbreviations: EC-ALB: elastic cerosomes loaded Albendazole, EE%: entrapment efficiency percent, and SAA: surfactant.

EE% of elastic cerosomes loaded Albendazole (EC-ALB) fluctuated from 66.52% ± 2.37% to 94.00% ± 3.58% (Table 3 and Figure 1). The resulting model equation in terms of coded factor was as follows:

$$\text{EE}\%=+75.72+0.82*X_{1}+1.04*X_{2}+0.017*X_{3}-5.13*X_{4}+0.98*X_{1}*X_{2}-2.77*X_{1}*X_{3}-2.80*X_{1}*X_{4}-0.49*X_{2}*X_{3}+3.44*X_{2}*X_{4}+2.90*X_{3}*X_{4}+12.41*X_{1}^{2}-8.81*\;X_{2}^{2}.$$

**TABLE 3:** The Composition and measured responses of the prepared EC-ALB according to the D-optimal design.

Formula code	X_1_ SAA amount (mg)	X_2_ Sonication time (min)	X_3_ Ceramide type	X_4_ Pluronic type	EE%^a^	PS (nm)^a^	PDI^a^	ZP^a^ (mV)
F1	75	30	IIIB	P 188	92.32 ± 1.98	639.10 ± 9.13	0.44 ± 0.13	−11.60 ± 0.01
F2	75	10	IIIB	P 188	94.00 ± 3.58	730.00 ± 60.01	0.67 ± 0.11	−16.50 ± 0.10
F3	50	20	IIIB	P 188	89.84 ± 5.95	645.00 ± 35.00	0.77 ± 0.06	−19.80 ± 0.02
F4	75	30	IIIB	P 188	92.32 ± 1.20	639.10 ± 11.73	0.441 ± 0.16	−16.90 ± 0.18
F5	62.5	15	IIIB	P 188	83.00 ± 8.83	597.30 ± 29.50	0.57 ± 0.07	−15.50 ± 0.04
F6	50	10	III	P 188	84.90 ± 1.54	669.90 ± 60.53	0.81 ± 0.12	−14.50 ± 0.22
F7	75	20	III	P 188	91.72 ± 1.05	668.20 ± 7.60	0.46 ± 0.17	−22.80 ± 0.12
F8	50	30	III	P 188	76.71 ± 1.76	740.00 ± 16.66	0.76 ± 0.02	−13.2 ± 0.680
F9	56.25	20	III	P 188	80.00 ± 0.96	659.00 ± 53.15	0.46 ± 0.11	−29.70 ± 0.33
F10	50	30	IIIB	L 121	74.10 ± 2.45	273.90 ± 7.90	0.49 ± 0.01	−24.60 ± 0.02
F11	50	10	IIIB	L 121	66.52 ± 2.37	335.30 ± 23.55	0.65 ± 0.06	−25.10 ± 0.90
F12	50	10	IIIB	L 121	66.52 ± 2.45	335.30 ± 23.55	0.64 ± 0.06	−26.30 ± 0.70
F13	75	20	IIIB	L 121	81.05 ± 2.30	384.10 ± 22.56	0.61 ± 0.05	−13.50 ± 0.15
F14	75	20	IIIB	L 121	81.05 ± 2.30	384.10 ± 2.30	0.60 ± 0.05	−13.30 ± 0.02
F15	62.5	30	III	L 121	70.00 ± 0.86	325.50 ± 27.87	0.55 ± 0.01	−14.00 ± 2.00
F16	50	20	III	L 121	91.01 ± 0.68	334.20 ± 1.56	0.40 ± 0.12	−13.80 ± 0.21
F17	62.5	15	III	L 121	68.00 ± 3.06	294.70 ± 10.25	0.54 ± 0.06	−14.50 ± 1.50
F18	75	30	III	L 121	76.41 ± 2.55	344.00 ± 40.07	0.52 ± 0.08	−13.80 ± 0.21
F19	75	10	III	L 121	68.00 ± 1.30	363.30 ± 9.06	0.62 ± 0.04	−13.50 ± 0.50

Abbreviations: ALB; albendazole, SAA, amount, surface active agent amount; EE%, entrapment efficiency percent; PS, particle size; PDI, polydispersity index, and EC-ALB, elastic cerosomes loaded albendazole.

^a^ Data represented as mean ± SD (n = 3).

Clear interaction had been observed between SAA amount (X_1_) and SAA type (X_4_) (P < 0.0001).

### 3.2 Effect of formulation variables on PS of elastic cerosomes loaded albendazole (EC-ALB)

**FIGURE 2:**
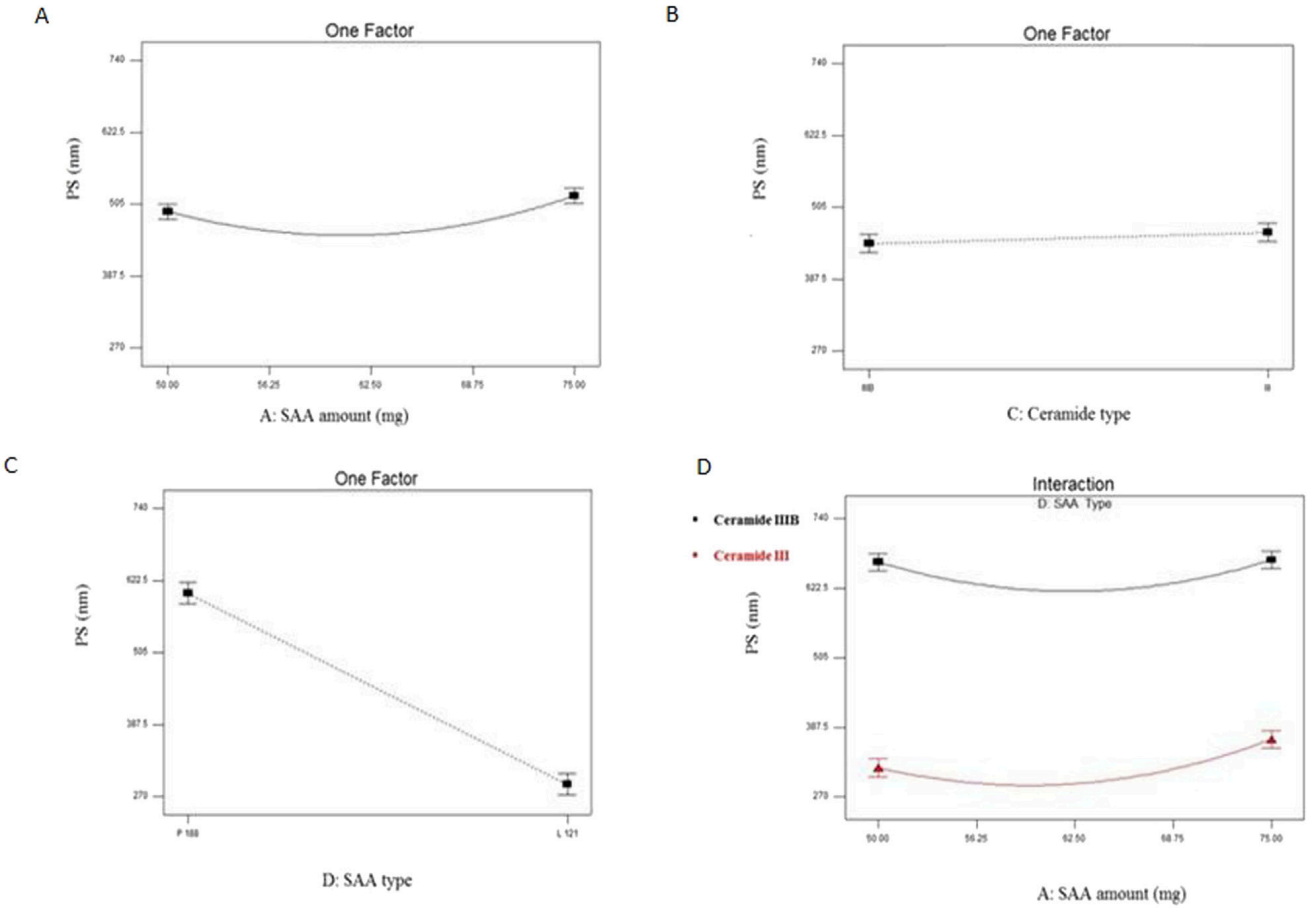
Line plots for the significant effect of SAA amount (X_1_) **(A)**, ceramide type (X_3_) **(B)** SAA type (X_4_) **(C)** and interaction plot between SAA amount (X_1_) and SAA type (X_4_) **(D)** on PS of EC-ALB. Abbreviations: EC-ALB: elastic cerosomes loaded Albendazole, PS: particle size, and SAA: surfactant.

PS values of all EC-ALB were in nano range (273.9 ± 7.90 to 740 ± 16.66 nm) as shown in Table 3 and Figure 2. The resulting model equation in terms of coded factor was as follows:

$$\text{PS }\left(\text{nm}\right)=+454.86+12.82*\;X_{1}-9.87*\;X_{2}+9.06*\;X_{3}-162.47*\;X_{4}-14.75*\;X_{1}\;*\;X_{2}-18.35*\;X_{1}\;*\;X_{3}+11.00\;*\;X_{1}*X_{4}+25.89*X_{2}*X_{3}-7.23*X_{2}*X_{4}-6.50*X_{3}*X_{4}+51.11*X_{1}^{2}+7.32*X_{2}^{2}.$$

SAA amount (X_1_), ceramide type (X_3_) and SAA type (X_4_) significantly affected the PS of EC-ALB (P = 0.0143 for SAA amount, 0.0358 for ceramide type and ˂0.0001 for SAA type).

### 3.3 Effect of formulation variables on PDI and ZP of elastic cerosomes loaded albendazole (EC-ALB)

**FIGURE 3:**
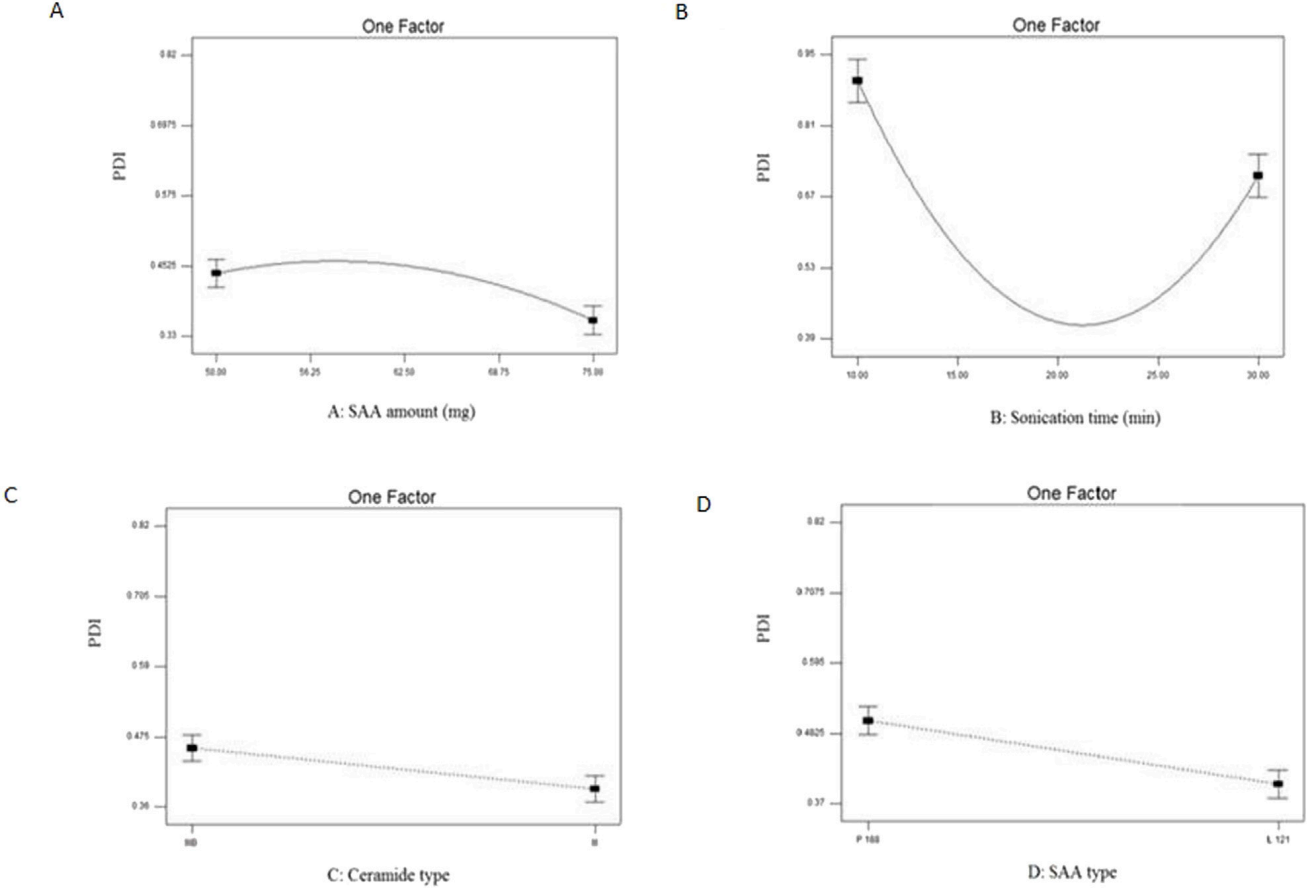
Line plots for the significant effect of SAA amount (X_1_) **(A)**, sonication time (X_2_) **(B)**, ceramide type (X_3_) **(C)** and SAA type (X_4_) **(D)** on PDI on EC-ALB. Abbreviations: EC-ALB: elastic cerosomes loaded Albendazole, PDI: polydispersity index, and SAA: surfactant.

PDI of EC-ALB ranged from 0.40 ± 0.12 to 0.81 ± 0.12 (Table 3). Some of EC-ALB were highly polydisperse, e.g., F3 (0.77 ± 0.06), F6 (0.81 ± 0.12) and F8 (0.76 ± 0.02) (Figure 3). The resulting model equation in terms of coded factor was as follows:

$$\text{PDI}=+0.45-0.041*X_{1}-0.070*X_{2}-0.035*X_{3}-0.053*X_{4}-0.051*X_{1}*X_{2}+3.688*10^{-3}*X_{1}*X_{3}+0.096*X_{1}*X_{4}+0.031*X_{2}*X_{3}-0.18*X_{2}*X_{4}-0.016*X_{3}*X_{4}-0.055*X_{1}^{2}+0.3*X_{2}^{2}+0.18*X_{1}*X_{2}*X_{3}-0.16*X_{1}*X_{3}*X_{4}+0.035*X_{2}*X_{3}*X_{4}.$$

All four variables significantly affected the PDI of EC-ALB (P = 0.010 for SAA amount, 0.0003 for sonication time, 0.0016 for ceramide type and 0.0005 for SAA type).

Considering ZP, All the prepared formulation are negatively charged and ranged from −11.60 ± 0.01 to −26.30 ± 0.70 mV.

### 3.4 Optimization of elastic cerosomes loaded albendazole (EC-ALB)

The total desirability of the optimal EC-ALB was 0.862 and it was suggested to be prepared using ceramide III (15 mg) and 50 mg Pluronic L121 with sonication time of 19.41 min. The formula was prepared and evaluated. Table 4 showed the small residual between the observed and predicted responses of the optimal EC-ALB which confirmed the rationality of the optimization process. Thus, optimal EC-ALB formulation was guaranteed to be used as a nucleus for preparing (SA-EC-ALB).

**TABLE 4:** Expected and observed values for the optimal EC-ALB.

Factor	Optimal level
X_1_: SAA amount (mg)	50
X_2_: Sonication time (min)	19.41
X_3_: Ceramide type	Ceramide III
X_4_: SAA type	Pluronic L121

Response	Expected	Observed	Residual^a^
Y_1_: EE%	90.46	92.03	−5.55
Y_2_: PS (nm)	339.23	312.05	5.03

^a^ Residual = Expected - Observed.

Abbreviations: EE%, entrapment efficiency percent, PS, particle size, EC-ALB, elastic cerosomes loaded albendazole.

### 3.5 Preparing of SA-EC-ALB using different amounts of SA

SA–EC–ALB formulations were developed with respect to EE% PS and ZP and examined to determine how positive charge inducers impact the physicochemical attributes of EC–ALB. As shown in Table 5, SA-EC-ALB prepared using 5 mg SA showed significantly higher EE% compared to those prepared using higher amounts of SA. Considering PS, SA-EC-ALB prepared using 10 and 15 mg were significantly larger than 5 mg SA-EC-ALB. Cerosomes prepared using 5 mg SAA showed ZP values of 38.2 ± 1.65 mV.

**TABLE 5:** The measured responses (EE% and PS) of SA-EC-ALB.

Amount of SA (mg)	EE%^a^	PS (nm)^a^	ZP (mV)^a^
5	92.13 ± 1.57	173 ± 52.33	38.2 ± 1.65
10	75 ± 4.24	386.5 ± 16.26	46.23 ± 0.93
15	71.85 ± 2.62	408.5 ± 4.95	56.63 ± 3.67

Abbreviations: SA, stearyl amine; EE %, entrapment efficiency percent; PS, particle size, ZP, zeta potential, and SA-EC-ALB, stearyl amine-elastic cerosomes loaded albendazole.

^a^ Data represented as mean ± SD (n = 3).

### 3.6 *In-vitro* release

**FIGURE 4:**
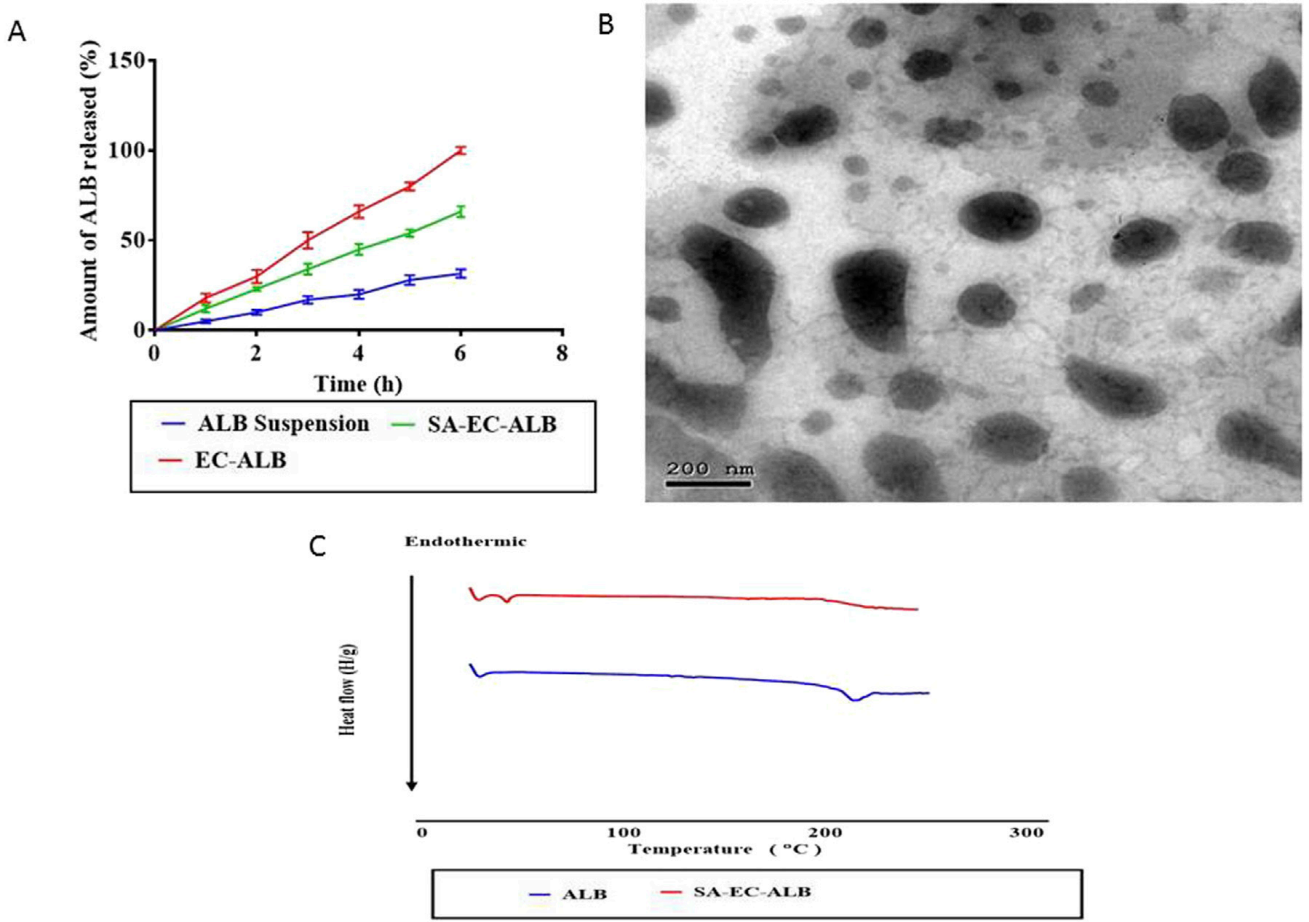
*In-vitro* release profile of SA-EC-ALB in comparison with ALB aqueous suspension. Data expressed as mean values ±standard deviation (n = 3) **(A)** and transmission electron micrograph of SA-EC-ALB **(B)**. Abbreviations: SA-EC-ALB: stearyl amine-elastic cerosomes loaded Albendazole, and ALB, Albendazole. **(C)** DSC study.

The *in-vitro* release profile of ALB from SA-EC-ALB and EC-ALB compared to ALB aqueous suspension is shown in Figure 4A. The release rate of ALB from SA–EC–ALB and EC–ALB formulations was significantly higher compared to the drug suspension. Furthermore, the extent of ALB released from SA-EC-ALB and EC-ALB after 6 h was significantly higher than ALB aqueous suspension.

### 3.7 Transmission electron microscopy

As shown in Figure 4B, SA-EC-ALB displayed elongated fiber like vesicles. Spherical vesicles occasionally appeared together with tubular ones.

### 3.8 Differential scanning calorimetry

Figure 4C represents the thermograms of pure ALB and optimal ALB elastic cerosomes. The DSC scan of pure ALB showed sharp endothermic peak at 204 °C corresponding to its melting point (Trandafirescu et al., 2019). This sharp peak disappeared in the thermogram of the optimal ALB elastic cerosomes.

### 3.9 Molecular docking studies

#### 3.9.1 *In silico* study of SA-EC-ALB binding affinity and thermodynamic stability

**FIGURE 5:**
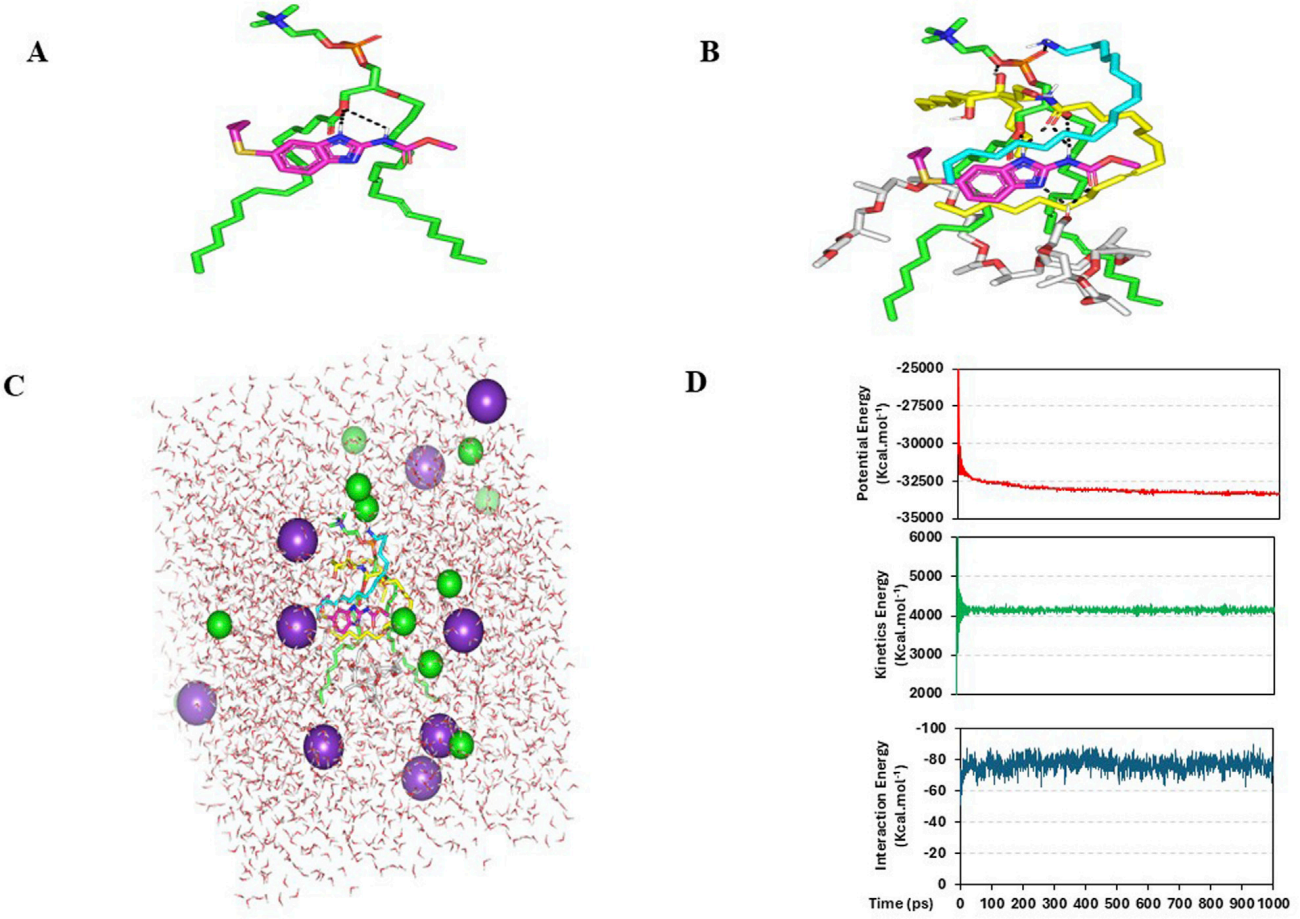
Predicted binding modes of ALB-PC docked complex. 3-D representation of ALB (magenta sticks) loaded on phospholipid interface (green sticks), alone **(A)** and in combination **(B)** with formulation additives; SA (cyan sticks), ceramide-III (yellow sticks), and Pluronic-L121 (white sticks). Described polar interactions are represented as black dashed-lines. SA-EC-ALB throughout all-atom molecular dynamics simulation at 100% aqueous solvation system. Solvated SA-EC-ALB within water cube and ionizable potassium and chloride atoms **(C)**. Plots for the system’s potential and kinetic energies, as well as the drug’s binding-free energy (upper, middle, and lower panels, respectively) versus the simulated time frames (ps) **(D)**.

ALB demonstrated a preferred orientation, aligning its aromatic core toward the acyl chains of PC and positioning it near the ester group. Further ALB-PC stability was highlighted through double polar interaction between the PC’s acyl ester oxygen and albendazole’s NH atoms of the benzimidazole ring and side arm substitution (Figure 5A). These hydrogen bonds were within favorable geometrical parameters, with distances of 2.81 Å and 3.02 Å, and bond angles of 134.20° and 122.30°, respectively.

**FIGURE 6:**
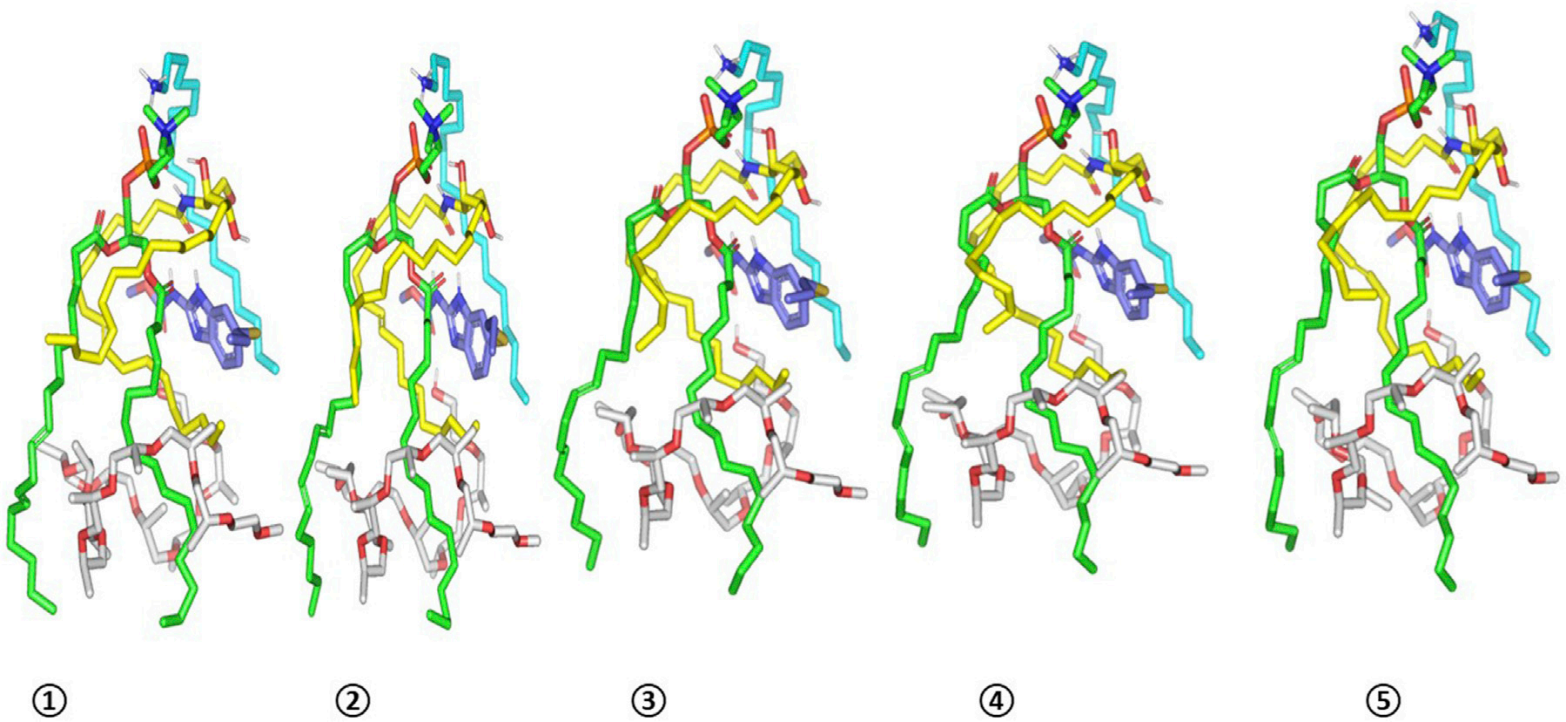
Conformation alterations-time evolution of SA-EC-ALB heterocomplex. Thermodynamic movements formulation components (sticks and differentially colored as previously described) were monitored over simulation trajectories being captured at different snapshots ① 200 ps, ② 400 ps, ③ 600 ps, ④ 800 ps, and ⑤ 1,000 ps.

ALB-PC complex in presence of the formulation additives is shown in Figure 5B. Dispersion behaviors and thermodynamic stability of ALB-PC nano-formulation complex within the final solvent of the formulation (100% water) were assessed through molecular dynamics simulations (Figure 5C). The system maintained a relaxed and equilibrated state, as indicated by consistent kinetic and potential energy profiles over the simulation period (Figure 5D). Analysis of conformation at extracted time intervals of 200, 400, 600, 800, and 1,000 ps is shown in (Figure 6).

### 3.10 Mucoadhesive evaluation of SA-EC-ALB

ZP of mucin suspension was found to be −8.47 ± 0.13 mV and exhibited changes upon mixing with mucoadhesive SA-EC-ALB. Mucoadhesive SA-EC-ALB showed a shift from negative mucin values −8.47 ± 0.13 mV to positive 15.00 ± 1.00 mV of mucin ZP value.

### 3.11 *In-vivo* studies

#### 3.11.1 Effect of ALB aqueous suspension and SA-EC-ALB on tumor volume

**FIGURE 7:**
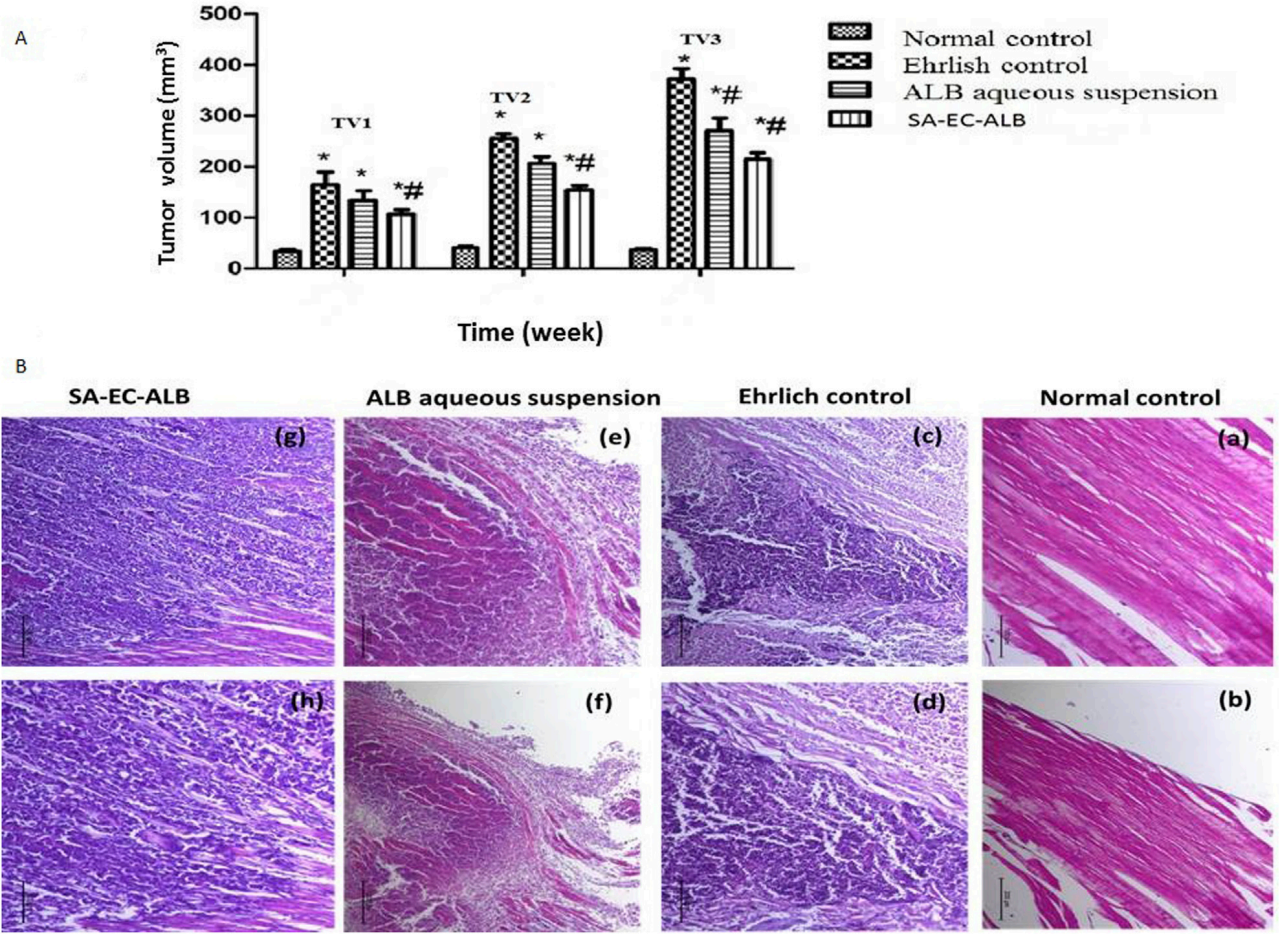
The effect of Albendazole and stearyl amine-elastic cerosomes loaded with Albendazole (SA-EC-ALB) on the tumor volume at different time intervals: TV1 at the end of first week, TV2 at the end of second week, and TV3 at the end of third week. (*) a significant difference from normal control, (#) a significant difference from Ehrlich control. **(A)** Photomicrographs showing histopathological sections stained with hematoxylin and eosin of Ehrlich tumor (n = 3 for each group, scale bar 100 and 200 nm for left and right panels, respectively). **(B)** a,b: normal control; c,d: Ehrlich control; e, f: animals treated with the Albendazole; g, h: animals treated with SA-EC-ALB. Abbreviations: SA-EC-ALB: stearyl amine-elastic cerosomes loaded Albendazole, and TV, tumor volume.

Tumor volumes (TVs) of the different groups were measured at different time intervals, at the end of first week (TV1), at the end of second week (TV2) and finally at the end of third week (TV3). At the three times, the Ehrlich control group revealed a marked enlargement of thigh volume compared to normal mice. For the group treated with ALB aqueous suspension, they did not show a significant difference from Ehrlich control except at the end of the third week (TV3), they showed a significant decrease in TV in comparison to that of Ehrlich control. Interestingly, the group treated with SA-EC-ALB showed a significant decrease (P<0.05) in TV as compared to Ehrlich control in the three time intervals (Figure 7A).

#### 3.11.2 *In-vivo* histopathological studies

A semi-quantitative histopathological scoring system was applied to evaluate the severity of necrosis, and inflammatory cell infiltration. Each parameter was scored on a scale from 0 to 3, where 0 = absent, 1 = mild, 2 = moderate, and 3 = severe. This scoring approach allowed for a comparative assessment of the pathological changes across different treatment groups.

As shown in Figure 7B, the histopathological examination of normal control mice thigh muscular tissues revealed the normal histological structure of striated muscle bundles with interstitium in between (score: 0 for all parameters). However, the mice of Ehrlich control showed a massive infiltration of tumor cells within the muscular tissues in focal and diffuse manner. Groups of pleomorphic large round and polygonal cells with hyperchromatic nuclei and binucleation were observed (necrosis: 1; inflammation: 3). Mice treated with ALB aqueous suspension presented a considerable degree of necrobiotic and atrophic changes with moderate inflammation within Ehrlich tumor cells, the muscle fibers were necrotic and atrophied in the most mice (necrosis: 2; inflammation: 2). Interestingly, despite the significant tumor volume reduction seen *in vivo*, the SA-EC-ALB group showed similar infiltration of neoplastic cells with little necrobiotic changes, and some involved in muscle fibers (necrosis: 1; inflammation: 3).

## 4 Discussion

### 4.1 Statistical design analysis

D-optimal design was selected in this study as its criterion depends on maximizing the determinant value of the information matrix to minimize the overall variance associated with estimating the model coefficients (Eldeeb et al., 2019). Furthermore; in D-optimal design, the software selected factorial points, axial check points, center of edges and overall center points (Shamma et al., 2012). These points’ diversity helps in providing a complete knowledge of responses with small number of experimental runs. Hence, a 19 run, four-factor, two-level D-optimal design was used for characterization and optimization of EC-ALB using Design-Expert^®^ software. The measured responses of the prepared 19 EC-ALB are shown in Table 3.

Selection of the model that best fits the data is the first step in statistical analysis. Therefore, sequential model comparison was performed for each response and showed that quadratic model is significant for responses Y_1_ and Y_2_ which indicates the fitting of the model to the measured response. For Y_3_, cubic model was aliased since more experiments are needed to be performed to estimate the model term accurately (Al Hagbani et al., 2018). The predicted R^2^ and adjusted R^2^ values are preferred to be in a reasonable agreement to confirm the fitting of the selected model to the data (Aziz et al., 2022a). Referring to design analysis results (Supplementary Table S1), it is clearly noticed that predicted R^2^ values are in good harmony with adjusted R^2^ values in all responses except PDI. Therefore, the variability in PDI might be attributed to noise (Al Hagbani et al., 2018) Furthermore, signal to noise ratio is measured by calculating adequate precision to make sure that the selected model can be used for design space evaluation (Aziz et al., 2022a). Adequate precision with a ratio greater than 4 is preferable and it was observed in all responses as shown in Supplementary Table S1.

### 4.2 Effect of formulation variables on EE% of elastic cerosomes loaded albendazole (EC-ALB)

Elastic cerosomes loaded Albendazole (EC-ALB) prepared using lower concentration of Pluronic L121 (SAA amount, X_1_) showed higher EE% compared to those prepared using higher SAA concentration. On the other hand, Pluronic P188 based vesicles showed increased EE% by increasing Pluronic P188 amount. This could be related to the higher hydrophilicity of Pluronic P188 (HLB = 29) compared to L121 (HLB = 1). Kadam Y. et al., reported that micelles prepared using hydrophobic polymers (higher % of PPO units) showed decrease in hydrodynamic size at higher concentrations with resultant decrease in the amount of solubilized drug (Kadam et al., 2009). In contrast, the hydrophilic Pluronics (e.g., P188) oppositely showed increase in micellar size with solubilized drug at higher concentration.

With respect to sonication time (X_2_), ANOVA results showed that EE% of EC-ALB prepared using sonication time 10 and 30 min was significantly lower than those prepared using intermediate sonication periods (15 and 20 min). In another words, it is noticed that EE% gradually increased by increasing the sonication time from 10 to 20 min. This might be attributed to that by increasing the sonication time in a suitable range, the number of vesicles per unit volume increased with consequent augmentation of the hydrophobic ambiance of the lipid bilayer to incorporate more hydrophobic drug (ALB) with resultant increase in EE%. These findings came in agreement with that presented by He Y. et al., who showed that the highest EE% of Ibuprofen liposomes achieved at the intermediate sonication time (20 min) (He et al., 2019). On the other hand, EE% significantly decreased by further increase in sonication time (X_4_) (30 min) as the too long sonication time might cause phospholipid degradation and disruption of the EC-ALB structures with resultant drug leakage and low EE% (Gala et al., 2015).

### 4.3 Effect of formulation variables on PS of elastic cerosomes loaded albendazole (EC-ALB)

With respect to SAA amount (X_1_), it was clearly noticed that the mean diameter of the prepared EC-ALB significantly decreased by initial increasing in SAA amount from 50 to 62.5 mg. This could be explained by the ability of SAA at higher levels to decrease the interfacial tension of the prepared nanosystem and increase the vesicular curvature with resultant decrease in PS (Albash et al., 2021). Furthermore; SAA, when used in sufficient amount, can increase the system stability by forming a steric barrier on its surface that prevents particles’ aggregation (Yousry et al., 2016) In contrast, PS significantly increased by further increasing in SAA amount (68.75 and 75 mg). This can be attributed to depletion-flocculation mechanism of SAA. In addition, SAAs beyond certain extent, form micelles in continuous phase rather than orientation on particles’ surface with resultant diminution of continuous phase between particles which consequently caused their aggregation and PS increase (Hasani et al., 2015).

With respect to ceramide type (X_3_); ceramide III-derived cerosomes were significantly larger in diameter than those prepared using ceramide IIIB. The major differences between both ceramides is that ceramide IIIB has one unsaturated bond in its fatty acid chain and with lower molecular weight (581.95) compared to ceramide III (583.98). Unsaturated ceramide molecules with lower molecular weight have more mobility to migrate between membrane leaflets compared to saturated larger ones which accumulated in the leaflets with consequent alteration in membrane curvature and increasing PS (Hasani et al., 2015; Albash et al., 2021).

Considering SAA type (X_4_), ANOVA results also showed that EC-ALB prepared using Pluronic P188 were significantly larger than those prepared using Pluronic L121. This could be explained based on the higher hydrophilicity of Pluronic P188 compared to L121 which consequently increases water uptake and causes PS enlargement (Aziz et al., 2022a).

### 4.4 Effect of formulation variables on PDI of elastic cerosomes loaded albendazole (EC-ALB)

PDI values of the prepared nano-formulation reflect the width of their size distribution which fluctuated from 0 (homogenous dispersion) to 1 (highly polydisperse particles with wide PS range) (Younes et al., 2018a). The results indicated that all EC-ALB formulations are homogenous with adequate PS distribution (Elsayed et al., 2020). The high PDI values of F3, F6 and F8 might be attributed to the irregularity of EC-ALB vesicular shape (Albash et al., 2021). Therefore, these systems were excluded in the optimization step.

With respect to SAA amount (X_1_), EC-ALB prepared using the highest amount of SAA (75 mg) were homogenously dispersed compared to those prepared using lower concentrations of SAA. This was attributed to the ability of Pluronics in lipid phase to induce steric stabilization which hinders particles’ aggregation (Younes et al., 2018a).

Considering sonication time (X_2_), ANOVA results showed that EC-ALB prepared using intermediate sonication periods (15 and 20 min) showed relatively more uniform particle distribution than those prepared using extreme sonication periods (10 and 30 min). As previously mentioned under EE% section, utilizing intermediate sonication periods increased the internalization of ALB within the formed EC-ALB and therefore, less free ALB would be available to form aggregates (Younes et al., 2018b).

For ceramide type (X_3_), EC-ALB prepared using ceramide III were relatively more homogenously dispersed than ceramide III-B -based EC-ALB. This could be attributed to the relatively smaller mean diameter of ceramide III-B-based vesicles. As a result, more vesicles per unit volume will be formed with consequent reduction in the external phase water volume level. Hence, less nucleation sites would be available for drug solubilization with resultant increased drug precipitation and increased PDI values (Nour et al., 2016).

Considering SAA type (X_4_), PDI values of EC-ALB prepared using Pluronic L121 were smaller than Pluronic P188-based EC-ALB. This could be explained based on the difference in their Mwt. Pluronic P188 showed higher Mwt (8,400 Da) compared to Pluronic L121 (4,400 Da) which caused less kinetically restricted entrapment of the drug with consequent increase in PDI (Younes et al., 2018b).

### 4.5 Preparing of SA-EC-ALB using different amounts of SA

The higher EE% of SA-EC-ALB prepared using 5 mg SA might be attributed to that, increasing positive charge within lipid bilayer results in changing the lateral packing of the vesicular bilayer and consequently decreasing EE% (Villasmil-Sánchez et al., 2010). Furthermore, it displayed smaller PS compared to other formulation. This can be interpreted by that increasing the amount of charge inducer increased the repulsive force between the vesicular adjacent bilayers with resultant increase in the spacing between them and formation of relatively large vesicles (Naguib and Makhlouf, 2021). With respect to vesicular charge (ZP), it was shown that ZP values ˃ 20 mV indicate an acceptable colloidal stability due to the presence of sufficient electrical charge on the nano-vesicular surface that could induce electrostatic repulsion between particles and prevent their aggregation. Furthermore, SA-EC-ALB prepared using 5 mg SA showed acceptable ZP values. Therefore, it was selected for further characterization.

### 4.6 *In-vitro* release

The higher rate and extent of ALB release from SA-EC-ALB compared to ALB aqueous suspension might be related to the solubilization of ALB by the phospholipid based-vesicular carrier (van Hoogevest et al., 2011). The phospholipid’s surface-active properties are widely recognized for enhancing the solubility of the poorly water-soluble drug enclosed within (Hills, 2002) On the other hand, the presence of SAA (Pluronic), might aid in the micelles formation within the bilayer which consequently may increase the membrane permeability and augments drug release (Albash et al., 2019).

### 4.7 Transmission electron microscopy

TEM imaging is valuable for describing the shape of the prepared system as well as for confirming the results of Malvern zetasizer (Abd-Elsalam et al., 2018b). Elongated fibers appeared due to ceramide partitioning into PC bilayer which consequently caused flattening of the vesicular bilayer curvature upon preparation. The occasional appearance of spherical vesicles together with tubular ones could be related to the un-even distribution of ceramide III in the vesicular bilayers which consequently caused the appearance of flattened ceramide rich domains together with spherical ceramide poor ones (Abdelgawad et al., 2017; Albash et al., 2021).

### 4.8 Differential scanning calorimetry

DSC is a valuable tool for investigating the potential phase changes and the drug-excipient interaction upon ALB encapsulation. The disappearance of the sharp endothermic peak of ALB in the thermogram of the optimal ALB- elastic cerosomes confirmed ALB transformation from crystalline to amorphous form due to its complete dispersion in the prepared nanosystem (Aziz et al., 2022a).

### 4.9 Molecular docking studies

#### 4.9.1 *In silico* study of SA-EC-ALB binding affinity and thermodynamic stability

Molecular docking study was conducted for exploring the extent and nature of interaction for ALB with the adopted formulation additives. Via the *in silico* study, ALB-PC interaction nature, in absence of other formulation additives, was primarily governed by van der Waal forces.

The observed binding mode of the compound with PC and its favored positioning near the phosphate head aligns with previous studies on small molecules with drug-like properties such as rosuvastatin, spironolactone, metformin, and levocetirizine, which have been investigated for their affinity towards phospholipid molecules (Abd-Elsalam et al., 2018a; Albash et al., 2021; Farag et al., 2021; Albash et al., 2022; Albash et al., 2023). Although this interaction was relevant, the docking results indicated modest binding energy (−2.27 kcal·mol^−1^), suggesting that additional stabilization would be beneficial for the ALB–PC complex.

The inclusion of formulation additives enhanced the ALB–PC complex, resulting in stronger and more stable binding interactions. Owing to the extended conformation of ceramide-III, this formulation additive depicted extended orientation around ALB-PC binding complex mediating several favored interactions. The amidic carbonyl of ceramide-III predicted strong hydrogen bonding with NH atoms of ALB (2.29 Å/141.33° and 2.26 Å/148.71°), while as its free hydroxyl group depicted favored polar interaction with PC phosphate group at 2.91 Å/128.09°. On the other hand, the extended hydrophobic arms of ceramide-III endorsed the aromatic scaffold of ALB as well as predicted favoured orientation towards the PC acyl arms (∼3.56 Å). Stability of ALB-PC was further mediated via SA as the latter showed strong hydrogen bond interaction with ALB polar head as well as close-distance hydrophobic contacts via its aliphatic chain with heterocyclic ring of ALB (∼2.64 Å). Notably, both the lipophilic aliphatic tails of ceramide-III and SA depicted a sandwich-like orientation around the docked ALB structure. Finally, the docked Pluronic L121 predicted a favored orientation near the PC acyl arms owing to its methyl branching extending from the polymeric chains. Nevertheless, the terminal hydroxyl group at Pluronic L121 unit depicted strong double polar interactions with polar functionalities of ALB including the carbonyl side chain and tertiary aromatic nitrogen (2.26 Å/134.01° and 2.35 Å/145.65°, respectively).

It is worth noting that PC and the two formulation additives (ceramide-III and SA) mediated double polar interactions (salt bridging) with polar functionalities of ALB serving as hydrogen bond donor and/or acceptor. Examination of all possible docking orientations revealed that incorporating the four formulation components resulted in a more stable ALB–PC complex, characterized by an extended network of hydrophobic and electrostatic interactions. This stabilization was reflected by an improved docking energy of −7.27 kcal·mol^−1^ for ALB within the nanoformulation complex. This enhanced binding may explain the improved formulation characteristics observed upon the addition of these additives, which act as carrier agents facilitating ALB loading onto the PC molecule and optimizing its solubilization.

The observed minimal energy fluctuations, maintain a plateau for over half the MD run indicate sufficient system stability and convergence. Conformational analysis at 200, 400, 600, 800, and 1000 ps demonstrated consistent stability of the simulated ALB–PC nanoformulation complex, with the drug retaining its position near the phospholipid acyl chains throughout the simulation (RMSD < 2.00 Å) Furthermore, ALB exhibited a considerably large negative free-binding energy (average ΔG = −76.81 ± 4.2 kcal.mol^−1^) towards the formulation components ensuring sufficient stability of the nano-formulation complex (Supplementary Figure S2). Ceramide-III and formulation additives kept their enveloped orientation around both ALB and PC molecules. Both SA and Pluronic L121 provided non-polar binding support for the phospholipid acyl chains as well as the drug’s benzimidazole core scaffold.

Finally, interesting results were disclosed regarding the spatial conformation of the phospholipid in terms of its elongated lipophilic chains. Conserved polar contacts at the phosphate group caused phospholipid’s hydrophobic acyl tails to separate from each other. Such thermodynamic behavior depicted an open-compass conformational structure for the extended tails of phospholipid causing higher volumes with larger solvent-accessible surface areas. On the contrarily, smaller solvent-accessible surface areas were maintained along the simulation run since the phospholipid complex maintained several strong compact polar interactions at the phosphate polar head. This observed packing style enabled ALB-PC nano-formulation complex to adopt an inverted cone structure with maintained micellar configuration being previously reported with various small molecules (Supplementary Figure S21; Israelachvili, 2011; Wang and Chung, 2013).

### 4.10 Mucoadhesive evaluation of SA-EC-ALB

The assemblies arising from mucin and SA-EC-ALB yielded a positive value, this suggests that the positive charge of SA–EC–ALB bound to the mucin surface neutralized its negative charge. The results further indicated that an adhesive component with mucoadhesive characteristics can alter the surface properties of mucin (Teaima et al., 2022).

### 4.11 *In-vivo* studies

#### 4.11.1 Effect of ALB aqueous suspension and SA-EC-ALB on tumor volume

The significant decrease in TV in group treated with SA-EC-ALB compared to Ehrlich control is due to the impact of Pluronics and SA in enhancing ALB solubility and retention, respectively. Hence, more freely soluble ALB would be available to induce its anti-cancer effect (Aziz et al., 2023). Furthermore, the presence of ceramides in vesicular constructs synergistically increased the anti-cancer efficacy of SA-EC-ALB due to the ceramides’ ability to induce an innumerable tumor suppressive signaling pathways, e.g., autophagy, apoptosis, and necroptosis (Barth et al., 2011; Galadari et al., 2015). Therefore, formulating ALB in SA-EC-ALB successfully augmented its anti-cancer efficacy compared to the free ALB suspension.

#### 4.11.2 *In-vivo* histopathological studies

Treatment with ALB aqueous suspension resulted in notable necrobiotic and atrophic changes within the Ehrlich tumor cells, demonstrating a substantial cytotoxic action of the treatment against the tumor cells but also led to some damage to the muscular tissue. In contrast, mice treated with SA-EC-ALB showed similar tumor cell infiltration but with reduced necrobiotic changes. The muscle fibers showed less signs of atrophy. This suggests that SA-EC-ALB could potentially offer a more balanced therapeutic approach, targeting tumor cells while preserving muscle tissue integrity to a greater extent.

## 5 Conclusion

Drug repurposing for cancer treatment is favored over new drug development in terms of bypassing the high costs and long processing time. In the present work, ALB (repurposed anti-cancer drug) was formulated in elastic cerosomes using thin film hydration technique according to D-optimal design to study the effect of formulation variables on the vesicular characteristics and to suggest the optimal elastic cerosomes loaded Albendazole (EC-ALB). Stearyl amine (SA) elastic cerosomes were then fabricated by incorporating different amounts of SA in the EC constructs. SA-EC-ALB prepared using 5 mg SA showed relatively high EE%, small PS and acceptable ZP and was selected for further characterization. SA-EC-ALB showed significantly higher release rate and extent compared to ALB aqueous suspension. Furthermore, anti-tumor assessment using Ehrlich tumor and histopathological study confirmed the augmented anti-cancer effect of ALB when incorporated in SA-EC-ALB. While these findings suggest a potential for enhanced therapeutic performance, further pharmacokinetic and biodistribution studies are required to confirm any enhancement in systemic bioavailability. Therefore, SA-EC-ALB represents a promising nanocarrier for enhancing the anticancer potential of ALB, pending additional validation.

## Data Availability

The original contributions presented in the study are included in the article/Supplementary Material, further inquiries can be directed to the corresponding author.
