# Supplementary material for: Stearyl amine-modified elastic cerosomes for boosting the anti-cancer activity of albendazole
Source: Front Pharmacol. 2025 Sep 4;16:1595177. doi: 10.3389/fphar.2025.1595177 (PMC12443754; doi:10.3389/fphar.2025.1595177)
Supplement: Supplementary file 1 [file Supplementaryfile1.docx]

| **Response** | **Model** | **R^2^** | **Adjusted R^2^** | **Predicted R^2^** | **Adequate precision** | **P value** | **Significant factors** | **Non-significant factors** |
| --- | --- | --- | --- | --- | --- | --- | --- | --- |
| **EE%** | Quadratic | 0.996 | 0.9879 | 0.8320 | 31.566 | < 0.0001 | X_1_ (0.0380), X_2_ (0.0200), X_4_(<0.0001) | X_3_ (0.9519) |
| **PS** | Quadratic | 0.9981 | 0.9942 | 0.9314 | 43.094 | < 0.0001 | X_1_ (0.0143), X_3_ (0.0358), X_4_ (<0.0001) | X_2_ (0.0500) |
| **PDI** | Cubic | 0.9986 | 0.9916 | N/A | 40.547 | Aliased | X_1_ (0.010), X_2_ (0.0003), X_3_ (0.0016), X_4_ (0.0005) | ------------- |

Supplementary Material

# Supplementary Table

**Supplementary Table 1.** Output data of the D-optimal mixture design implemented for optimization of EC-ALB

**Abbreviations:** EE%, entrapment efficiency percent; PS, particle size, PDI, polydispersity index, and EC-ALB, elastic cerosomes loaded albendazole.

# Supplementary Figures


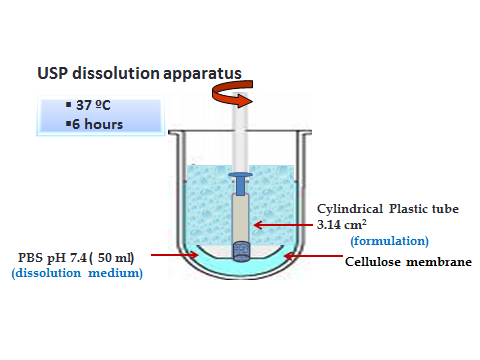


Supplementary **Figure 1.** Schematic illustration of the modified USP dissolution apparatus used for the in vitro release study.

**
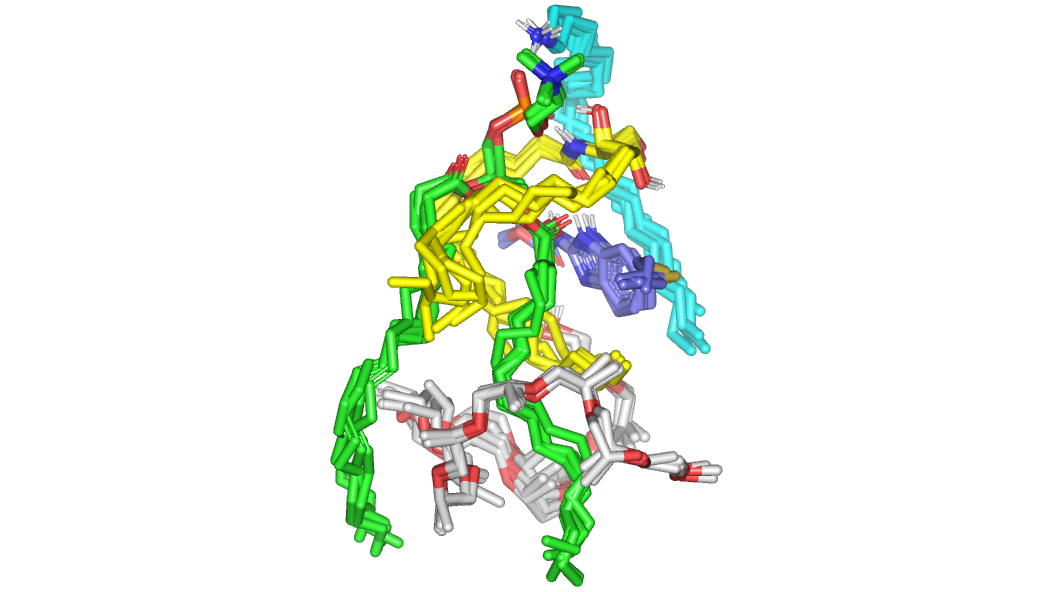
**
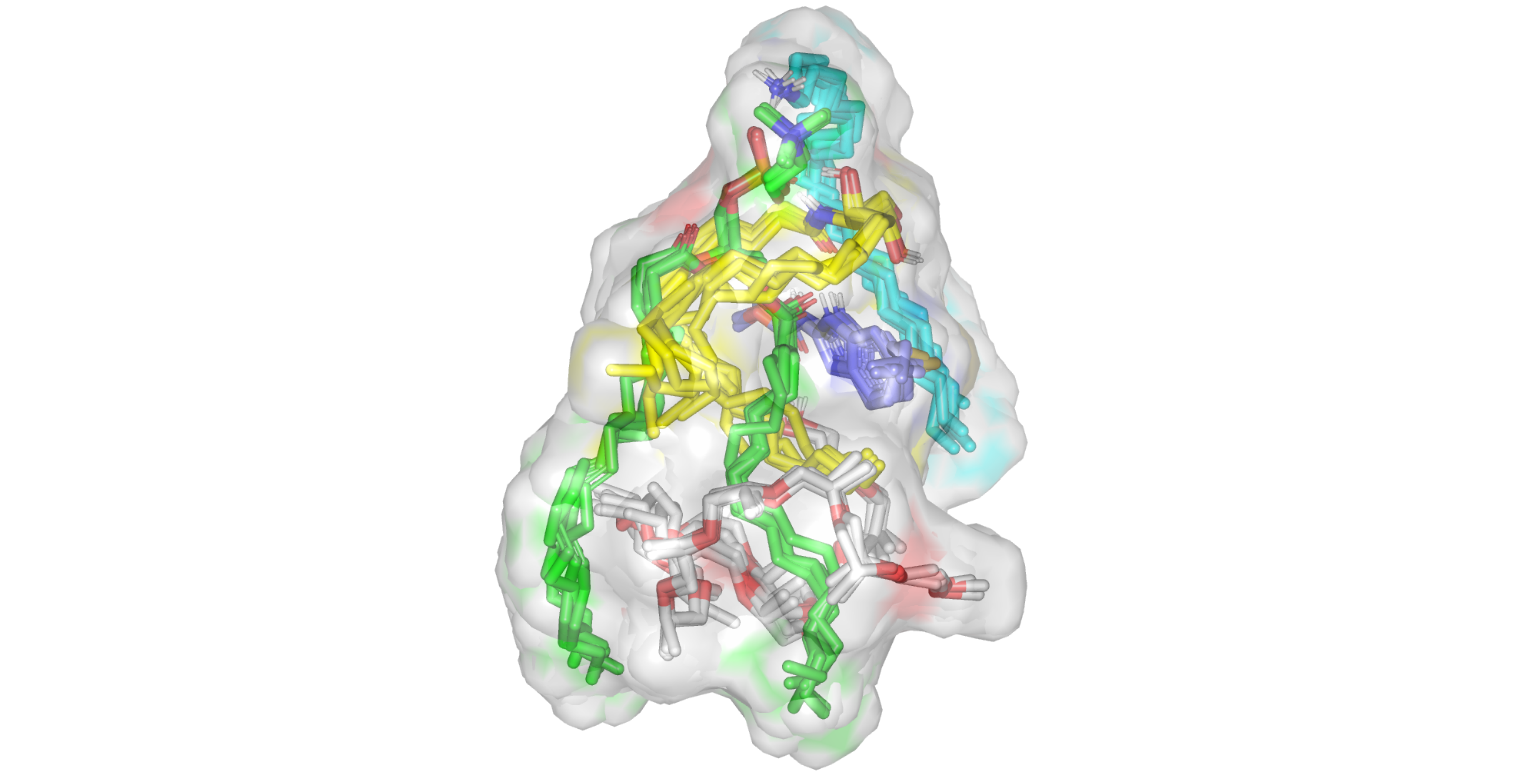


Supplementary Figure 2: Overlaid SA-EC-ALB heterocomplex across molecular simulation frames (left panel) and molecular surface 3D-representation of the inverted cone micellar configuration at 100% water solvation system (right panel). Molecular sticks and surface 3D-representations were illustrated in colors being previously assigned for the optimal formulation components .
